# Construction of Spleen-Accumulated Polysorbate 20-Containing Ionizable Lipid Nanoparticles for mRNA Delivery

**DOI:** 10.3390/nano15241844

**Published:** 2025-12-08

**Authors:** Hanyu Liu, Siqi Li, Kexin Chen, Shuyi Yao, Xuefeng Tang, Xiaojun Han

**Affiliations:** State Key Laboratory of Urban-Rural Water Resource and Environment, MIIT Key Laboratory of Critical Materials Technology for New Energy Conversion and Storage, Heilongjiang Provincial Joint Laboratory of Molecular Science (International Cooperation), School of Chemistry and Chemical Engineering, Harbin Institute of Technology, Harbin 150001, China; 2022111035@stu.hit.edu.cn (H.L.); 2022110638@stu.hit.edu.cn (K.C.); 2022112668@stu.hit.edu.cn (S.Y.)

**Keywords:** ionizable lipid nanoparticles, polysorbate, mRNA delivery, spleen-accumulated, drug design

## Abstract

Messenger RNA therapy represents a transformative therapeutic in vaccine development, tumor immunotherapy, and genetic disease intervention. Polyethylene glycol (PEG) lipid, a key component of ionizable lipid nanoparticles (iLNPs) for mRNA delivery, and the PEG antibodies induced by PEG are associated with hypersensitivity and accelerated blood clearance. To address the above PEG-associated challenges, we systematically investigated polysorbate 20 (PS20) as an alternative for constructing PEG-free iLNPs. The formulation of PS20-incorporated iLNPs (PS20-iLNPs) for carrying mRNA was systematically investigated by analyzing the particle size, pKa, endosomal escape efficiency, and cellular internalization efficiency of iLNPs with different molar ratio PS20 content (0.5–5.0%). iLNPs with a relative molar ratio of 2.5% were identified as the optimal mRNA delivery carrier. This carrier exhibited excellent resistance to serum protein adsorption capacity, with serum stability 1.3-fold higher than PEG-iLNPs. At high lipid concentrations (2.7 mg/mL), the cell viability of PS20-iLNPs was maintained at 91.1%, which was 1.07-fold higher than PEG-iLNPs. Under serum interference, PS20-iLNPs achieved a transfection efficiency of 46.5%, marking a 10.6% improvement over PEG-iLNPs under identical conditions. Notably, PS20-iLNPs exhibited 24 times higher spleen accumulation than PEG-iLNPs. These findings highlight PS20 as a viable PEG substitute for developing PEG-free spleen-accumulated mRNA delivery platforms with enhanced therapeutic potential.

## 1. Introduction

Messenger RNA (mRNA) therapy has been demonstrated as having considerable potential as a revolutionary therapeutic for the disease prevention, treatment, and vaccine development in recent years [1,2]. It can encode specific proteins for achieving precise intervention without the risk of genomic integration [3,4,5]. However, mRNA therapeutic applications remain constrained by inherent physicochemical barriers (hydrophilicity, electronegativity), structural bulkiness, and rapid degradation, collectively impeding intracellular delivery and translational efficiency [2,6]. These challenges may be addressed through engineered delivery systems, such as lipid-based vehicles [7,8], viral platforms [9,10], inorganic nanoparticles [11,12], and target delivery Luc-mRNA by one-component Janus dendrimers [13,14]. Among these, Ionizable lipid nanoparticles (iLNPs) have become a premier effective delivery carrier for mRNA vaccines, owing to their biocompatibility modifiable surface properties, and efficient encapsulation and delivery capabilities.

The canonical formulation of ionizable lipid nanoparticles (iLNPs) incorporates four structural constituents: ionizable lipids, cholesterol, phospholipids, and polyethylene glycolconjugated lipids (PEG-lipids) [15,16], each working synergistically to contribute to high-efficiency mRNA encapsulation, structural stability of iLNPs [17]. PEG-lipids, in particular, enhance nanoparticle homogeneity, structural stability, and hydrophilicity while reducing immune recognition and prolonging circulation time in vivo [18,19,20]. However, the immunogenicity, allergic reactions caused by lipid nanoparticles, as well as the diminished therapeutic efficacy observed upon repeated administration, are primarily attributed to PEGylated lipids [21,22,23]. These effects stem from the propensity of PEGylated lipids to interact with immune cells, thereby eliciting the production of undesirable antibodies, including anti-PEG antibodies (APA) [24,25,26]. Given the widespread presence of PEG in consumer products [27], PEG can rapidly induce elevated titers of anti-PEG antibodies (APA) within a few days [28]; therefore, preexisting APA is detectable in ~70% of the population, even in individuals who have never received PEGylated therapy [29,30]. The high homology between APA and selected germline sequences of B cells in most individuals further exacerbates APA occurrence [27]. Additionally, preexisting APA increases the risk of rapid-onset pseudoallergic reactions or other hyper-sensitivity reactions (HSR) [1,27,31,32], with severe cases potentially progressing to anaphylactic shock. At least three PEGylated therapeutics are currently withdrawn from clinical use in part due to severe HSR [33]. Furthermore, APA-mediated accelerated blood clearance (ABC) phenomenon reduces cellular uptake of therapeutics upon repeated administration, which, in turn, decreases therapeutic efficacy and increases the metabolic burden on the liver and kidney [34,]. Consequently, the development of suitable PEG substitutes has become an urgent need for the development of ionizable lipid-based drug delivery systems.

The emerging PEG alternatives, predominantly including polymeric architectures (e.g., poly(2-ethyl-2-oxazoline) (PEtOx) [35], poly(2-methyl-2oxazoline) (PMOZ) [36], poly(2-methacryloyloxyethyl phosphorylcholine) (PMPC) [37,38], brush-shaped poly(ethylene glycol) methyl ether methacrylate (PEGMA) [39] and polysarcosine (pSar) [40,41] and lipid derivatives (e.g., gangliosides) [42], have demonstrated feasibility in constructing next-generation PEG-free lipid nanoparticles (LNPs). These engineered PEG-free LNPs systems exhibited superior performance, particularly in stealthiness properties, antifouling ability, biocompatibility, and cellular transfection efficiency. Despite these advancements, the most current PEG-free LNPs maintained preferential hepatic accumulation, fundamentally constraining their therapeutic utility in extrahepatic pathologies. Polysorbate 20 (PS20), a nonionic surfactant with a molecular structure consisting of a hydrophilic polyoxyethylene head and hydrophobic fatty acid tail [43], exhibits excellent interfacial activity, enabling it to displace proteins from the oil–water interface while stabilizing the oil droplets against coalescence. Therefore, PS20 has been widely used in the food, cosmetics, and pharmaceutical fields to stabilize emulsions, prevent surface adsorption, and inhibit protein aggregation [44]. We postulate that PS20 can maintain nanoparticle stability and homogeneity, reduce protein adsorption and aggregation, and prolong blood circulation time. Moreover, PS20 may alter drug biodistribution in vivo by controlling the size of nanoparticles, which orchestrates size-dependent biodistribution remodeling to potentiate the extrahepatic delivery precision of drugs.

**Scheme 1:**
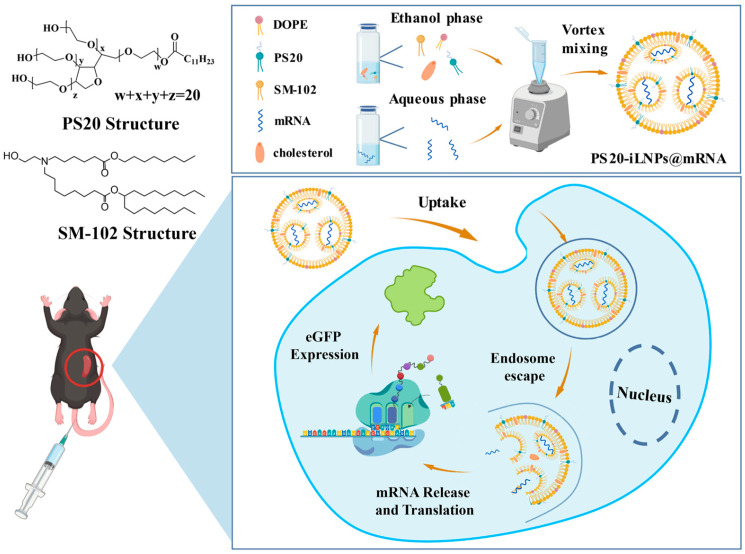
Scheme illustration of PS20-iLNPs encapsulating eGFP mRNA and their translation inside cells. The PS20-iLNPs@mRNA consist of SM-102, DOPE, cholesterol, PS20 and eGFP mRNA. After intravenous injection, iLNPs exhibit spleen-specific and highly efficient accumulation. Upon cellular uptake, eGFP mRNA is released and translated into green fluorescent proteins.

In this study, we developed a PS20-containing lipid nanoparticle with superior delivery and safety. As shown in Scheme 1, iLNPs was based on SM-102, it is an ionizable lipid that serves as a primary component of iLNPs, and it is crucial for nucleic acid encapsulation and endosomal escape. iLNPs loaded with mRNA were prepared via the vortex mixing method using SM-102, DOPE, cholesterol, PS20, and eGFP mRNA. After cellular internalization, the release of mRNA was hindered by endosomes. Progressive endosomal maturation induces protonation and a resultant positive charge on SM-102 ionizable lipids. This initiates electrostatic interactions with endosomal membrane lipids, triggering the formation of hemifusion pores and a phase change from lamellar to hexagonal HII phase, which collectively disrupt endosomal stability to facilitate the cytoplasmic release of eGFP mRNA for translation. Furthermore, following intravenous administration, PS20-iLNPs exhibited a distinct biodistribution profile, with significantly higher accumulation in the spleen compared to PEG-iLNPs. In contrast to PEG-iLNPs, PS20-iLNPs exhibited enhanced stealthiness properties, elevated transfection efficiency, and spleen accumulating capacity, with no observed increase in cell toxicity. This strategy effectively achieved extrahepatic accumulating of iLNPs.

## 2. Materials and Methods

### 2.1. Materials

SM-102 and DMG-PEG2000 were purchased from ShoChem (Shanghai, China) Co., Ltd. Poly-sorbate (PS) 20, 5-(N, N-hexamethylene) amiloride, 2-(p-toluidino)6-naphthalenesulfonic acid (TNS), 1,10dioctadecyl-3,3,30,30-tetramethylindotricarbocyanine iodide (DiR), and dialysis bags (with cutoff molecular weights (WMCO) 3 kDa) were purchased from Sigma-Aldrich (Shanghai, China). Cholesterol and DOPE were purchased from Avanti Polar Lipids (Alabaster, AL, USA). DMEM high glucose with L-glutamine, DNase/RNase-free distilled water and fetal bovine serum (FBS) were purchased from Thermo Fisher (Shanghai, China). FAM/Cy5-labelled mRNA were purchased from GenePharma (Suzhou, China). EGFP mRNA was purchased from APExBIO (Shanghai, China). Hoechst 33342 Staining Solution for Live Cells and Enhanced Cell Counting Kit-8 were purchased from Beyotime Biotechnology (Shanghai, China). Healthy female Balb/c mice (6–8 weeks old) were obtained from Beijing Vital River Laboratory Animal Technology Co., Ltd. (Beijing China).

### 2.2. Preparation of iLNPs by Vortex Mixing Method

PS20-iLNPs with varying molar ratios were prepared by the vortex mixing method according to the molar ratios detailed in Table S1. The lipid ethanol solution, which contained SM-102, cholesterol, DOPE, and PS20, was added to the pH 4 sodium citrate buffer at a volumetric ratio of 1:3 (lipid ethanol solution: citrate buffer) and vortexed rapidly. The resulting mixture was incubated at 37 °C for 1 h, followed by dialysis in sodium citrate buffer (pH 4) for 1 h and subsequently in PBS buffer (pH 7.4) for 3 h. PEG-iLNPs were synthesized using the same protocol but were incubated for 15 min at room temperature and dialyzed in PBS (pH 7.4) for at least 1 h.

iLNPs@mRNA were prepared via the vortex mixing method likewise. SM-102, cholesterol, DOPE, and PS20 were dissolved in ethanol at a molar ratio of 50%:37.5%:10%:2.5%, while Cy5 or FAM-labeled mRNA was dissolved in 10 mM sodium citrate buffer (pH 4). The solutions were quickly vortexed and mixed at a 1:3 (lipid ethanol solution: RNA citrate buffer) volumetric ratio. The resulting mixture was incubated at 37 °C for 1 h and dialyzed for 4 h.

### 2.3. iLNPs Characterization

The hydrodynamic size, polydispersity index (PDI), and zeta potential of the iLNPs were measured by dynamic light scattering (DLS, Zetasizer Nano ZEN5600, Malvern Instruments Limited, Malvern, UK). The mRNA encapsulation efficiency was evaluated using a fluorescence spectrometer. The morphology of PEG-iLNPs@eGFP mRNA and PS20-iLNPs@eGFP mRNA was observed using a transmission electron microscopy (TEM) (JEM-2100, JEOL, Tokyo, Japan) with an acceleration voltage of 200 kV. The iLNPs were deposited on a carbon-coated copper grid and dried under ambient conditions. Negative staining was performed by applying 10 μL of 1% phosphotungstic acid solution to the grid for 60 sec and then air-dried at room temperature.

### 2.4. Apparent pKa Analysis

The apparent pKa values of PS20-iLNPs were determined by 2-(p-toluidino)-6-napthalene sulfonic acid (TNS) fluorescent probe. PS20-iLNPs were prepared and stored in PBS with a concentration of 5 mM total lipids. The 100 μM TNS solution and a series of TNS buffer solutions with pH values from 3 to 10 were prepared. TNS buffer contained 1 μM TNS, 10 mM ammonium acetate, 10 mM morpholineethanesulfonic acid (MES), 10 mM HEPES and 130 mM NaCl. The above solutions were vortex mixed, and the fluorescence intensity was measured by spectrophotometer (Ex 321 nm, Em 445 nm) (Fluorescence spectrometer LS 55, PerkinElmer, Waltham, MA, USA) at room temperature. A sigmoidal best fit analysis was applied to the fluorescence data and the pKa was determined as the pH with half-maximal fluorescence intensity.

### 2.5. Membrane Disruption Capacity Experiment

Red blood cells (RBCs) were isolated from fresh whole blood collected from C57BL/6 mice via centrifugation at 500× *g* for 5 min. The isolated RBCs were subsequently washed with phosphate-buffered saline (PBS) buffer (pH 7.4) until the supernatant became clear. The RBCs were resuspended in PBS buffers of pH 7.4 and pH 5.5, respectively. PS20-iLNPs (50 μg) with varying molar ratios of PS20 were added to RBC suspension with an equal number of RBCs. After incubating at 37 °C for 2 h, the RBC solutions were centrifuged at 10,000× *g* for 5 min. The collected supernatants containing hemoglobin content were added to 96-well plates. The absorbance at 450 nm was measured to evaluate the hemoglobin using the microplate reader. The RBC suspension incubated in PBS buffer (pH 7.4) and Triton-100 (3 wt.%) solution served as negative and positive control, respectively.

A hemolysis assay was performed to evaluate the relationship between endosomal escape efficiency and iLNPs concentration. Different concentrations of iLNPs containing 2.5% PS20 were added to RBC suspension at 37 °C for 2 h. Hemoglobin content was assessed by measuring absorbance at 450 nm using a microplate reader.

### 2.6. Cellular Uptake of iLNPs with Different Ratio of PS20

The uptake of iLNPs containing different proportions of PS20 was analyzed by flow cytometry. C6 cells were seeded into 6 well cell culture plates with the density of 2 × 10^5^ cells per well and incubated for 24 h (37 °C, 5% CO_2_). The cells were incubated with different kinds of PS20-iLNPs@Cy5-mRNA (molar ratio of PS20 is 0.5%, 1%, 1.5%, 2.5%, 5%, respectively) for 4 h, which were detached with trypsin, harvested and resuspended in PBS for the flow cytometry assay and analyzed using FlowJo_V10.

### 2.7. Exploration of Optimal Ratio of mRNA to Lipid (N/P)

The FAM-labeled mRNA was used to prepare mRNA solutions with concentrations of 6.0 nmol/mL, 4.8 nmol/mL, 3.6 nmol/mL, 2.4 nmol/mL, 1.2 nmol/mL, and 0 nmol/mL. The fluorescence intensity of FAM-mRNA at 490 nm was measured using a fluorescence spectrophotometer to establish a calibration curve correlating fluorescence intensity with concentration. PS20-iLNPs@FAM-mRNA with N/P ratio of 2, 4, 6, 8 and 10 were prepared, and Triton-100 was used to break the membrane at 0 h, 24 h, 48 h and 72 h, respectively. Fluorescence spectrometer was used to detect the fluorescence intensity of each group iLNPs and calculate the encapsulation rate. To verify and analyze the stability, hydrodynamic sizes, PDI and zeta-potentials of iLNPs were measured by DLS.

### 2.8. Agarose Gel Electrophoresis

The PS20-iLNPs@mRNA were prepared as mentioned above. Equal concentrations of eGFP mRNA, PS20-iLNPs@eGFP mRNA, PS20-iLNPs@eGFP mRNA with Triton-100 were, respectively, combined with loading buffer (Solarbio, R1055, Beijing, China) and loaded in 2% agarose gel, whose electrophoresis in 1× TAE for 15 min. The results were detected using Amersham Imager 600 (GE Healthcare, Boston, MA, USA).

### 2.9. Stability of iLNPs

The time-dependent stability of PS20-iLNPs and PEG-iLNPs was assessed in the presence or absence of serum. PS20-iLNPs and PEG-iLNPs were added to PBS and PBS with 10% FBS (FBS-PBS), respectively. The iLNPs were diluted with PBS or FBS-PBS at a volume ratio of 1:9 (total lipid 2.5 mg/mL). The iLNPs were incubated at 37 °C for 0.5, 2, 24, 48, and 72 h, then measured by dynamic light scattering (DLS, Zetasizer Nano ZEN5600, Malvern, UK) to monitor the changes in hydrodynamic size, polydispersity index (PDI), and zeta-potential of the iLNPs.

### 2.10. Protein Adsorption Test

PS20-iLNPs and PEG-iLNPs were added to PBS and PBS with 10% (*v*/*v*) bovine serum albumin (BSA-PBS), and then incubated at 37 °C for 72 h. The hydrodynamic size (*Hs*) of the iLNPs were measured by DLS. The fraction of stealthiness (*Fs*) was calculated using below equation. *Fs* value close to 1 indicates that the iLNPs exhibit excellent stealth properties, whereas an *Fs* value approaching 0 signifies the instability of iLNPs due to the presence of proteins.

$$Fs=\frac{\left(Hs\;in\;PBS\right)}{\left(Hs\;in\;PBS\;with\;BSA\right)}$$

### 2.11. Safety of iLNPs

Cell viability was assessed using the CCK-8 assay. The C6 cells were seeded into 96-well plates at a density of 3 × 10^3^ cells per well and cultured for 24 h. Each well contained 10 μL of iLNPs and 90 μL of culture medium. PBS treated group was used as the control. After 24 h incubation, 10 μL CCK-8 solution was added to each well and further incubated for a period from 30 to 60 min. The optical density (OD) at 450 nm was measured by microplate reader (ELx-808, BioTek Instruments, Winooski, VT, USA). The cell viability is calculated using below equation.

$$Cell\;Viability=\frac{\left([(As-Ab)]\right)}{\left([(Ac-Ab)]\right)}\times 100\%$$

where *As* is the optical density of cells exposed to iLNPs, *Ac* is the optical density of PBS group, *Ab* is the optical density of wells with no cells.

### 2.12. Intracellular iLNPs@mRNA Delivery

The flow cytometry (BriCyte E6, Mindray, Shenzhen, China) and laser scanning confocal microscopy (LSCM, FV3000, Olympus, Tokyo, Japan) were used to detect intracellular uptake of PS20-iLNPs@Cy5-mRNA. C6 cells were seeded in 6-well plates at a density of 2 × 10^5^ cells per well and incubated for 24 h. After treating with PS20-iLNPs@Cy5-mRNA for 0, 1, 2, 4, 6, 8, and 12 h. The fluorescence signal was examined using flow cytometry (BriCyte E6, Mindray, Shenzhen, China) and analyzed with Flow Jo V10. C6 cells were seeded in cell-culture dishes (Applied Nest, Issaquah, WA, USA) and cultured for 24 h. PS20-iLNPs@Cy5-mRNA was added to the cell-culture dishes and treated for 0, 1, 2, 4, 6, 8, and 12 h. The images were acquired using an Olympus FV 3000 microscope (Tokyo, Japan) after the nuclei were stained with Hochest 33342.

C6 cells were seeded in 6-well plates at a density of 2 × 10^5^ cells per well and incubated for 24 h. After treating with different concentrations of PS20-iLNPs@Cy5-mRNA for 6 h. The uptake of Cy5-labeled mRNA delivered by iLNPs at different concentrations was detected by flow cytometry (Mindray, BriCyte E6) and assessed with Flow Jo V10. C6 cells were seeded in cell-culture dishes and cultured for 24 h. The cells were treated by adding different concentrations of PS20-iLNPs@Cy5-mRNA for 6 h. The nuclei were stained with Hochest 33342 for 15 min, washed three times with PBS, and images were collected using an Olympus FV 3000 microscope.

### 2.13. PS20-iLNPs@Cy5-mRNA Cell Internalization Pathway

C6 cells were seeded in 6-well plates at a density of 2 × 10^5^ cells per well and incubated at 37 °C with 5% CO_2_ for 24 h, and pretreated with serum-free medium containing inhibitors (sucrose at 450 mM, M-β-CD at 2 mM, and 5-(N-ethyl-N-isopropyl)-amiloride (EIPA) at 10 μg/mL) for 30 min. Subsequently, the cells were incubated with PS20-iLNPs@mRNA for 6 h. After the cells were digested with trypsin, harvested, and resuspended in PBS, the cellular uptake of PS20-iLNPs@Cy5-mRNA under different inhibitor conditions was examined using flow cytometry (Mindray, BriCyte E6) and analyzed with Flow Jo V10.

### 2.14. In Vitro Transfection Efficiency

The transfection efficiency of PEG-iLNPs@eGFP mRNA and PS20-iLNPs@eGFP mRNA was assessed using flow cytometry and laser scanning confocal microscopy (LSCM). C6 cells were seeded in 6-well plates at a density of 2 × 10^5^ cells per well and incubated for 24 h at 37 °C with 5% CO_2_. The cells were treated with iLNPs at a mRNA concentration of 30 μg/mL for 48 h. After trypsinization, the cells were harvested, resuspended in PBS, examined by flow cytometry (Mindray, BriCyte E6) and analyzed with Flow Jo V10. C6 cells were seeded in confocal dishes (Nest, Issaquah, WA, USA) and cultured for 24 h. PEG-iLNPs@eGFP mRNA and PS20-iLNPs@eGFP mRNA were added to the cell culture dishes and incubated for 48 h. Images were acquired using an Olympus FV 3000 microscope.

### 2.15. In Vivo Biodistribution

DiR was dissolved in methanol and then mixed into a phospholipid mixture solution dissolved in chloroform. After evaporating under a stream of nitrogen gas, an ethanol solution was added. The resulting mixture was vortexed with sodium citrate buffer solution at a 1:3 (*v*/*v*) ratio and incubated. Free DiR was removed by dialysis. DiR-tagged PS20-iLNPs and PEG-iLNPs were tail-vein injected into C57BL/6 mice (0.15 mg lipid per mice), respectively. The mice were sacrificed and heart, liver, spleen, lung, kidney were excised 6 h after the injection, respectively. Eventually, the fluorescence images were acquired by IVIS system (ABL-X5, Tanon, Shanghai, China) to analyze the average fluorescence intensity in these major organs.

### 2.16. Statistical Analysis

All datasets were statistically assessed using GraphPad Prism 10.1.2, with triplicate-independent experiments expressed as mean ± SD. Two-group comparisons employed Student’s *t*-test, whereas multiple comparisons utilized one-way ANOVA. Significance thresholds were defined as: * *p* < 0.05, ** *p* < 0.01, *** *p* < 0.001, and **** *p* < 0.0001.

## 3. Results

### 3.1. Synthesis and Characterization of PS20-iLNPs

**Figure 1:**
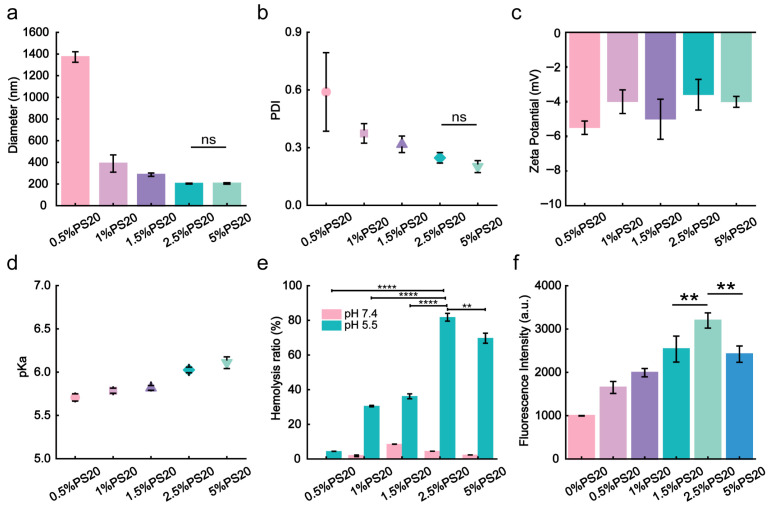
Screening, optimization, and characterization of PS20-iLNPs formulations. (**a**) Particle diameter of PS20-iLNPs. (**b**) Polydispersity index (PDI) of PS20-iLNPs in each group. (**c**) Zeta potential of PS20-iLNPs. (**d**) pKa values of PS20-iLNPs. Symbol legend: circle, 0.5% PS20 group; square, 1%; regular triangle, 1.5%; diamond, 2.5%; inverted triangle, 5%. (**e**). In vitro lysosomal escape efficiency of PS20-iLNPs. (**f**) Cellular uptake treated with PS20-iLNPs@Cy5-mRNA Data are expressed as mean ± s.d (n = 3 biologically independent samples). Statistical analyses were performed by two-tailed unpaired *t*-test and one-way ANOVA: ** *p* < 0.01, **** *p* < 0.0001, ns, not significant.

A series of PS20-containing iLNPs (molar ratios ranging from 0.5% to 30%) were prepared via vortex mixing method to optimize the formulation. To ensure endosomal escape of iLNPs, the molar ratios of SM-102 and DOPE were fixed, while cholesterol and PS20 molar ratios were modulated between 39.5 and 10.0% and 0.5–30%, respectively (Table S1). As shown in Figure 1a,b, the size and polydispersity index (PDI) of PS20-iLNPs gradually decreased with increasing PS20 relative molar ratios. At PS20 molar ratios of 1.5–5%, the size of PS20-iLNPs stabilized, while the PDI continued to decrease, implying that the presence of more PS20 enhanced the homogenization of nanoparticles. Conversely, the increase in the relative molar ratio of PS20 from 5% to 30% resulted in larger and heterogeneous nanoparticles (Figure S1). These results indicated that PS20, as an excipient, significantly influenced iLNPs size and homogeneity. The optimal PS20 concentrations range from 1.5% to 5%. iLNPs with varying concentrations of PS20 exhibited a slightly negative zeta potential (~−5 mV) (Figure 1c), enabling the resistance to serum protein adsorption [45], prolonging circulation. pKa value has been demonstrated to determine the surface charge property, which is believed to be related to the facilitated endosomal escape process [46]. The highly efficient intracellular delivery of bioactive macromolecules with optimal values is between 6.0 and 6.5 [47]. Therefore, 2.5% and 5% PS20-iLNPs likely exhibit optimal endosomal escape efficiency (Figure 1d and Figure S2). To validate this hypothesis, the membrane disruption capacity of PS20-iLNPs was quantified via a hemolysis assay. The results show that 2.5% and 5% PS20-iLNPs achieved endosomal escape efficiencies of 81.8% and 69.7%, respectively (Figure 1e). Notably, PS20-iLNPs exhibited negligible membrane disruption ability at physiological pH 7.4 relative to pH 5.5, indicating that iLNPs maintained structural integrity and safety in blood circulation. For RNA payload delivery optimization, formulation screening was conducted through flow cytometric quantification of cellular internalization. As shown in Figure 1f and Figure S3, Cy5-labeled mRNA fluorescence was detected in all groups after being treated with PS20-iLNPs (containing different relative molar ratios of PS20), while the highest fluorescence intensity appeared in the 2.5% PS20-iLNPs group. These findings suggest that 2.5% PS20-iLNPs represent an optimized drug delivery vehicle. For further studies, 2.5% PS20-iLNPs (molar ratio of SM-102, cholesterol, DOPE, and PS20 = 50:37.5:10:2.5) were selected for detailed characterization and mRNA delivery studies.

### 3.2. Stability and Stealthiness of PS20-iLNPs

**Figure 2:**
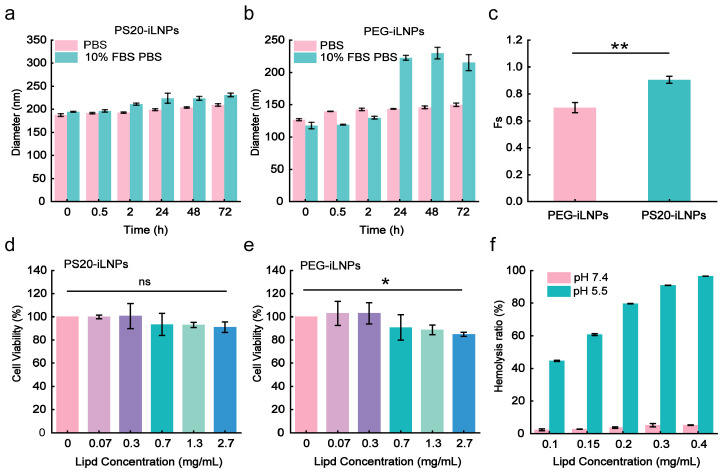
Stability, stealth capacity, and safety of PS20-iLNPs compared to PEG-iLNPs. (**a**,**b**) Particle diameter of both formulations measured by DLS under various incubation times and media conditions. (**c**) Fraction of stealthiness (*Fs*) of both formulations after incubation with serum-supplemented (BSA in PBS) and serum-free solutions (PBS). (**d**,**e**) Cell viability in different lipid concentrations of both formulations detected by CCK-8 assay. (**f**) Circulatory safety and in vitro lysosomal escape efficiency of different concentrations of PS20-iLNPs detected by hemolysis assay. Data are expressed as mean ± s.d (n = 3 biologically independent samples). Statistical analyses were performed by two-tailed unpaired *t*-test and one-way ANOVA: * *p* < 0.05, ** *p* < 0.01; ns, not significant.

The stability and stealthiness of the 2.5% PS20-iLNPs (referred to as PS20-iLNPs), were examined using PEG-iLNPs as the control group. After 72 h incubation in PBS with 10% fetal bovine serum (FBS), dynamic light scattering (DLS) results revealed a substantial size increase in PEG-iLNPs group from 117 nm to 215 nm (Figure 2b and Figure S4c,d), exhibiting an 82.6% growth rate, particularly evident at 24h with significant particle enlargement. In contrast, the PS20-iLNPs group exhibited a size increase from 194 nm to 231 nm (Figure 2a and Figure S4a,b), with a growth rate of 19.1%, indicating their superior stability compared to PEG-iLNPs. The anti-protein corona capability of PS20-iLNPs was assessed through adding BSA into the PBS and incubating with iLNPs under physiological conditions (pH 7.0, 37 °C). The hydrodynamic size (*Hs*) of protein adsorption on both iLNPs were expressed as the fraction of stealthiness (*Fs*) values. *Fs* value close to 1 indicated that iLNPs possess excellent stealthiness, whereas *Fs* value approaching to 0 suggests instability in the presence of proteins. As shown in Figure S5, both formulations demonstrated comparable stealth capacity within the initial 24 h, while from 24 to 72 h, PS20-iLNPs exhibited significantly higher *Fs* values than PEG-iLNPs (Figure S5 and Figure 2c), highlighting PS20’s superior structural stabilization and protein corona mitigation capabilities. PS20-iLNPs exhibited stealth characteristics comparable to those of the zwitterionic polymer poly(2-methacryloyloxyethyl phosphorylcholine) (PMPC) as a PEG substitute [37], representing PS20 had the potential to alternative polyethylene glycol (PEG) in biomedical applications.

### 3.3. In Vitro Safety

Safety is a crucial consideration in drug delivery carrier development, as it is directly related to the clinical application potential and patient treatment safety. iLNPs were co-incubated with cells for 24 h, and the safety of PEG-iLNPs and PS20-iLNPs was examined by CCK-8 assay. As shown in Figure 2d,e, both iLNPs showed no toxicity in C6 cells at lower lipid concentrations (0–0.3 mg/mL). At high lipid concentration (0.7–2.7 mg/mL), the cell viability of PS20-iLNPs group exhibited a negligible decrease. Even at the highest concentration (2.7 mg/mL), the cell viability remained at 91.1%, with no observable morphological changes. In contrast, the cell viability of the PEG-iLNPs group was significantly decreased. These findings demonstrate PS20-iLNPs’ enhanced biocompatibility relative to PEG-iLNPs across lipid dosage gradients, suggesting dose-dependent therapeutic efficacy optimization through controlled PS20-iLNP administration.

Additionally, we employed a hemolysis assay to evaluate the safety of PS20-iLNPs during blood circulation and to simulate their endosomal escape efficiency in cells. As shown in Figure 2f, erythrocyte hemolysis was minimal in the physiological environment (pH 7.4), demonstrating that PS20-iLNPs can effectively maintain the structural integrity and ensure the safety during blood circulation. It is noteworthy that under the acidic environment (pH 5.5), the hemolysis rate gradually increased with the increase in PS20-iLNPs concentration, suggesting efficient endosomal escape of iLNPs in a concentration-dependent manner. These findings support the potential of PS20-iLNPs for efficient cellular uptake and transfection.

### 3.4. Delivery Capacity of PS20-iLNPs@Cy5mRNA In Vitro

**Figure 3:**
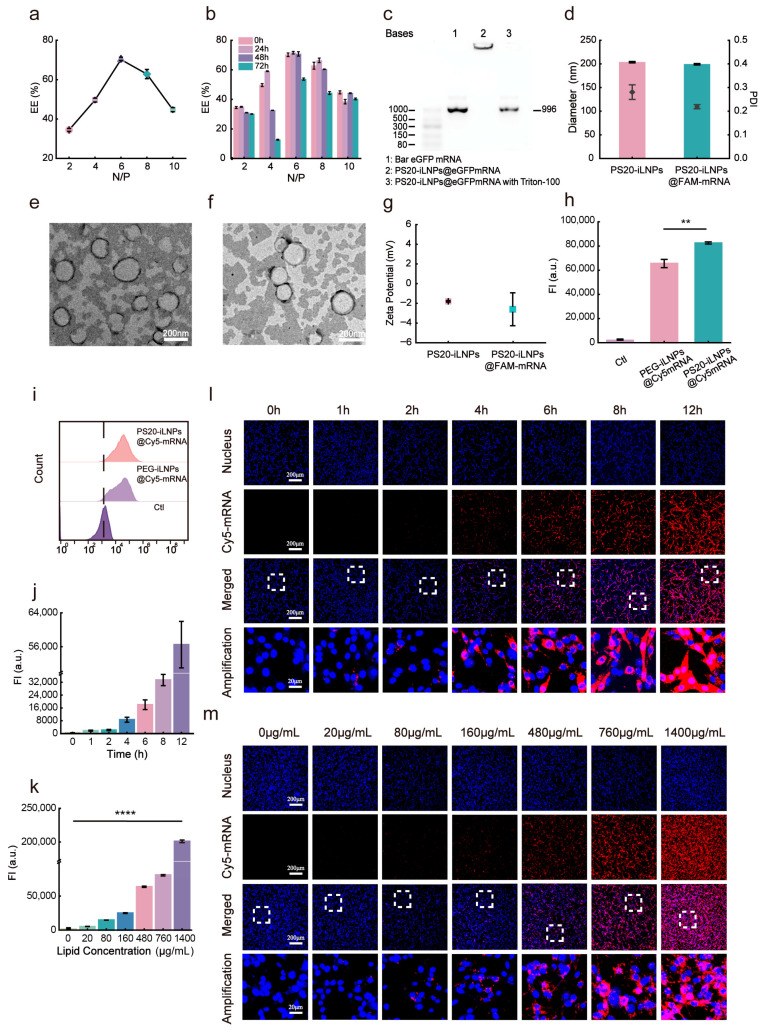
Cellular uptake efficiency and mechanism of PS20-iLNPs@mRNA. (**a**) Encapsulation efficiency of PS20-iLNPs@mRNA at different N/P ratios. (**b**) Variation in the PS20-iLNPs@mRNA encapsulation efficiency at different N/P from 0 to 72 h. (**c**) Agarose gel electrophoresis of bare eGFP mRNA, PS20-iLNPs@eGFP mRNA without and with Triton-100. (**d**) Comparison of particle diameter and PDI of PS20-iLNPs (N/P = 6 for PS20-iLNPs@mRNA) between loaded and unloaded mRNA (**e**) TEM image of PS20-iLNPs@eGFP mRNA. (**f**) TEM image of PEG-iLNPs@eGFP mRNA. (**g**) Comparison of Zeta-Potential PS20-iLNPs (N/P = 6 for PS20-iLNPs@mRNA) between loaded and unloaded mRNA. (**h**,**i**) Delivery efficiency of Cy5-mRNA into C6 cells by PEG-iLNPs and PS20-iLNPs, as detected by flow cytometry. Flow cytometry (**j**) and confocal imaging (**l**) assessed the time-dependent cellular uptake of PS20-iLNPs@mRNA by C6 cells. Flow cytometry (**k**) and confocal imaging (**m**) assessed the dose-dependent cellular uptake of PS20-iLNPs@mRNA by C6 cells. The area within the dashed box in l and m is shown at higher magnification in the panel below. Scale bars: 200 μm and 20 μm. Data are expressed as mean ± s.d. (n = 3 biologically independent samples). Statistical analyses were analyzed using two-tailed unpaired *t*-test and one-way ANOVA: ** *p* < 0.01, **** *p* < 0.0001.

Ionizable lipid-mRNA lipid nanoparticles are formed through electrostatic interactions between the ionizable amine groups of lipid-like polyamines and the phosphate groups of mRNA molecules. The N/P ratio, defined as the molar ratio of nitrogen (in the amine head-groups of lipopolyamines) to phosphorus (in the oligonucleotide backbones), is a critical parameter for evaluating nucleic acid delivery efficiency using lipid nanoparticle [48]. This study aimed to determine the optimal N/P ratio for encapsulating Cy5-labeled mRNA in PS20-iLNPs (PS20-iLNPs@Cy5mRNA). As shown in Figure 3a, the highest encapsulation efficiency (EE%) of 70.2% was at N/P ratio of 6. The PS20-iLNPs@Cy5mRNA at varying N/P ratios were stored at 4 °C for 72 h, the encapsulation efficiency of iLNPs decreased in all groups except for N/P ratio of 6 group (Figure 3b and Figure S6), which indicated that the interactions between the phospholipids and the nucleic acids were the most stable at N/P of 6, ensuring the structural stability of the PS20-iLNPs@Cy5mRNA. Agarose gel electrophoresis analysis demonstrated that eGFP mRNA was effectively encapsulated within PS20-iLNPs@eGFP mRNA, with maintained mRNA integrity (Figure 3c). Further physical characterization revealed that PS20-iLNPs@Cy5mRNA had an average diameter of 199.3 nm. Electrostatically driven interaction between negatively charged mRNA and protonated SM-102 resulted in slightly reduced particle size of PS20-iLNPs@Cy5mRNA than that of bare PS20-iLNPs (Figure 3d). The morphological features of PS20-iLNPs@eGFP mRNA and PEG-iLNPs@eGFP mRNA were visualized using transmission electron microscopy (TEM), demonstrating nanoparticles with near-spherical topography and well-defined peripheral boundaries. (Figure 3e,f). Under physiological pH, both loaded and bare PS20-iLNPs exhibited near-neutral zeta-potentials (−2.4467 mV), demonstrating that the mRNA was successfully encapsulated in iLNPs (Figure 3g).

Given the critical role of efficient intracellular uptake and delivery of PS20-iLNPs in genetic therapy, the cellular internalization of PS20-iLNPs@Cy5mRNA and PEG-iLNPs@Cy5mRNA were quantitatively analyzed by flow cytometry. We incubated both iLNPs with the C6 cells for 4 h in the presence of serum. PS20-iLNPs@Cy5mRNA showed a 25.9% increase in mean fluorescence intensity (82,526 vs. 65,545) compared to PEG-iLNPs@Cy5mRNA (Figure 3h,i). This may be attributed to the superior anti-protein adsorption capability of PS20-iLNPs (consistent with Figure 2c), which reduces protein corona formation to enhance cellular uptake.

To elucidate the mRNA delivery mechanism of PS20-iLNPs, we investigated the cellular uptake patterns and pathways of the optimal formulation PS20-iLNP. Using Cy5-labeled mRNA, we observed that PS20-iLNPs@Cy5mRNA was significantly internalized by cells after 4 h incubation (Figure 3l). Furthermore, the uptake efficiency increased with prolonged incubation time (Figure 3j and Figure S7b). Based on these findings, we fixed the interaction time of cells-iLNPs at 6 h. After a 6 h incubation in same amount cells with varying iLNPs concentrations, the minimum concentration for effective cellular uptake of PS20-iLNPs@Cy5mRNA was determined to be 80 μg/mL (Figure 3m). Intracellular fluorescence intensity (FI) increased with higher administration concentrations, confirming concentration-dependent cellular uptake (Figure 3k and Figure S7a). Next, we investigated the endocytic pathways using sucrose, EIPA and M-β-CD, which are inhibitors to clathrin-mediated endocytosis, macropinocytosis and caveolae-mediated endocytosis, respectively. For PEG-iLNPs, M-β-CD and sucrose significantly reduced cellular uptake by 70.4% and 61.5%, respectively (Figure S8a,b), suggesting that caveolae-mediated and clathrin-mediated endocytosis are the primary pathways. However, in PS20-iLNPs group, EIPA and sucrose inhibited the cellular uptake efficiency by 46.2% and 20%, respectively (Figure S8c,d), which indicated that PS20-iLNPs are different from PEG-iLNPs; they are mainly internalized via macropinocytosis, and lesser via clathrin-mediated endocytosis. Despite employing distinct internalization pathways, the two iLNP formulations exhibit comparable intracellular distribution after entry (Figure S9).

### 3.5. In Vitro eGFPmRNA Transfection

**Figure 4:**
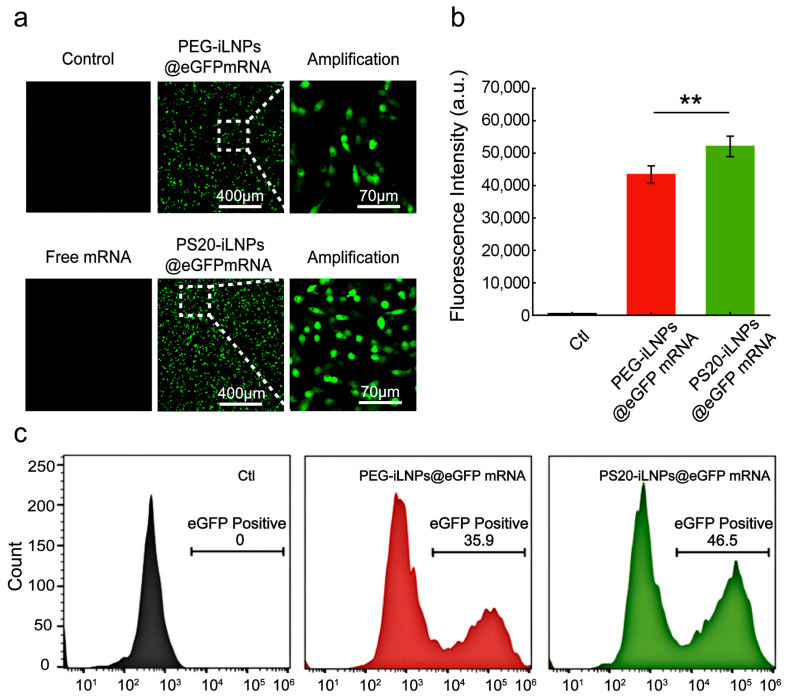
Intracellular transfection of PEG-iLNPs@eGFPmRNA and PS20-iLNPs@eGFPmRNA. (**a**) Laser scanning confocal microscopy images of C6 cells transfected with both formulations. Scale bars: 400 μm and 70 μm. (**b**) Flow cytometry detection of eGFP fluorescence intensity in C6 cells treated with both formulations. (**c**) Transfection efficiency of both formulations analyzed by FlowJo V10. Data are expressed as mean ± standard deviation (mean ± s.d). (n = 3). Statistical analyses were analyzed using two-tailed unpaired *t*-test: ** *p* < 0.01.

The transfection efficiency was evaluated using the expression levels of eGFP mRNA encapsulated in PEG-iLNPs (PEG-iLNPs@eGFP mRNA) and PS20-iLNPs (PS20-iLNPs@eGFP mRNA). After transfecting C6 cells for 48 h, the expression of eGFP mRNA was assessed by confocal microscopy and flow cytometry. As shown in Figure 4a, confocal microscopy revealed that both PS20 and PEG group can effectively deliver eGFP mRNA into cells, preserving mRNA biological function and enabling successful expression. Figure S10 indicated that the transfection ability of PS20-iLNPs was higher than PEG-iLNPs by analyzing the fluorescence signals of Figure 4a using ImageJ. Subsequently, the delivery efficiency of both iLNPs was further quantified by flow cytometry. Figure 4b showed that the eGFP expression in PS20 group was slightly higher than PEG group. Quantitative analysis showed that the mean eGFP fluorescence intensities of PEG and PS20 group were 43,388 and 52,075, respectively, which were consistent with the trend of Figure S10. The transfection efficiency of PS20 group was 46.5%, which was 10.6% higher than that of PEG group (Figure 4c). Polysarcosine (pSar) has been demonstrated to serve as a polyethylene glycol (PEG) alternative for lipid nanoparticle (LNP) formulation [40]. However, the mRNA expression with the pSar systems was lower than with the PEG-based lipid nanoparticle [40]. PS20-iLNPs achieved superior transfection performance than PEG-iLNPs, which indicated the enhanced efficacy of PS20 over pSar as the PEG substitute in iLNPs gene delivery systems.

Collectively, these data establish PS20 as a multifunctional excipient that inhibits protein corona formulation, improves biocompatibility, and facilitates efficient mRNA delivery, leading to functional protein translation. This formulation represents a viable PEG-free strategy for advanced nanoparticle engineering.

### 3.6. In Vivo Biodistribution of PS20-iLNPs

**Figure 5:**
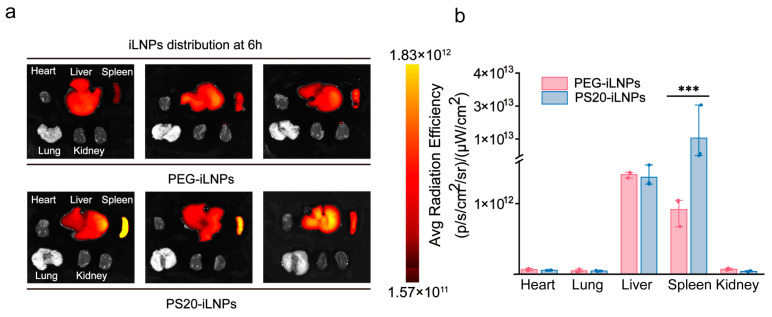
In vivo biodistribution of PEG-iLNPs and PS20-iLNPs. Mice were intravenously injected with DiR-labeled PEG-iLNPs and PS20-iLNPs via the tail vein. Fluorescence images of major organs (**a**) and quantitative analysis (**b**) were acquired at 6 h post-injection. Data are expressed as mean ± standard deviation (mean ± s.d). (n = 3 biologically independent samples). Statistical significance was analyzed using two-tailed unpaired *t*-tests: *** *p* < 0.001.

Given the significance of systemic mRNA delivery in numerous applications, including protein replacement therapy and systemic immunization, we further investigated the distribution characteristics of PS20-iLNPs drug delivery system in animals following systemic injection. DiR-labeled iLNPs were intravenously injected into C57BL/6 mice via the tail vein, and the mice were sacrificed 6 h post-injection for isolation and examination of major organs. The fluorescence signals of isolated organs were recorded by in vivo imaging system to monitor the distribution of PS20-iLNPs. The result of fluorescence signals indicated that iLNPs were mainly accumulated in the liver and spleen (Figure 5a). Notably, the fluorescence intensity statistical analysis exhibited that PS20-iLNPs possess significantly higher infiltration to spleen than PEG-iLNPs (Figure 5b), with approximately 24-fold higher accumulation. While the gangliosides-based LNPs developed by Permana et al. exhibited stealth properties and protein resistance comparable to PEG-based LNPs, their biodistribution demonstrated dual hepatic/splenic accumulation, which could potentially exacerbate drug-induced hepatotoxicity [42]. In contrast, PS20-iLNPs achieved spleen-specific accumulation without elevating hepatic metabolic burden, maintaining safety with precision delivery. The splenic accumulation mechanism of PS20-iLNPs is primarily attributed to their optimized particle size (199.3 ± 1.5 nm). This dimension aligns with the structural features of splenic venous sinusoids, where the reticuloendothelial cell gaps are approximately 200–500 nm [49]. Numerous studies have demonstrated that nanoparticles within the 200–500 nm range exhibit preferential splenic retention due to mechanical filtration mechanisms [50,51,52,53]. Moreover, Zhang et al. conducted comparative analyses revealing that 215 nm nanoparticles achieved superior splenic enrichment relative to 165 nm and 285 nm counterparts, thereby establishing 200 nm as an optimal threshold for efficient splenic targeting [54]. On the other hand, owing to the constitutive activity of macropinocytosis in immature dendritic cells (DCs), particles of relatively larger sized (e.g., ≥200 nm) are advantageous for DC targeting [55,56,57]. Our PS20-iLNPs size of ~200 nm may explain the spleen accumulate property (Figure 5).

## 4. Conclusions

Despite the significant success of phospholipid-based drug delivery systems in the field of nucleic acid delivery, it is still a challenge to overcome the side effects induced by the excipient PEG in the formulations of LNPs. We optimized PS20-contained ionizable lipid nanoparticles (PS20-iLNPs) to identify 2.5% PS20 containing LNPs (PDI < 0.3, encapsulation efficiency > 70%). The PS20-iLNPs retained superior stability and stealth properties (Δsize ≈ 36.7 nm) versus PEG-iLNPs (Δsize ≈ 97.3 nm) during 72 h storage and showed excellent safety (cell viability > 90%) at 2.7 mg/mL lipid concentrations. The time-/concentration-dependent internalization kinetics indicated that macropinocytosis played the main role, with partial contribution from clathrin-mediated endocytosis. The transfection efficiency of 46.5% under serum conditions is 10.6% improvement over PEG systems, indicating the better mRNA delivery function of PS20-iLNPs. The biodistribution revealed that PS20-iLNPs significantly accumulated in the spleen, being 24-fold higher than PEG-iLNPs. PS20-iLNPs offer a dual-functional platform that combines PEG-free safety with precision spleen accumulating capabilities, providing a promising drug delivery platform for the cancer, viral infections, inflammation, and autoimmune diseases treatment.

## Data Availability

Data are contained within the article and Supplementary Materials.

## Supplementary Materials

The following supporting information can be downloaded at: https://www.mdpi.com/article/10.3390/nano15241844/s1, Table S1. Formulation of iLNPs with diverse molar ratio of PS20; Figure S1. Size distribution of 5% (a), 10% (b), 15% (c), and 30% (d) PS20-iLNPs detected by DLS. Figure S2. TNS assay. Fluorescence values of 0.5% (a), 1.0% (b), 1.5% (c), 2.5% (d), 5.0% (e) PS20-iLNPs (0.5–5.0%) at different pH values and S-type fitting curves. Figure S3. Flow cytometry of C6 cells incubated with iLNPs containing different concentrations of PS20 for 4 h. Figure S4. The PDI (a,c) and Zeta-potential (b,d) of PS20-iLNPs and PEG-iLNPs with different incubation periods characterized by DLS. Figure S5. Fs value of PS20-iLNPs (a) and PEG-iLNPs (b) with different incubation periods. Figure S6. Encapsulation efficiency of PS20-iLNPs@mRNA at different N/P ratios after 24 h (a), 48 h (b) and 72 h (c). (d) The calibration curve of TNS assay. (e) The size and PDI of PS20-iLNPs@mRNA at different N/P ratios. (f) The zeta-potential of PS20-iLNPs@mRNA at different N/P ratios. Figure S7. Flow cytometry of C6 cells incubated with iLNPs to assess dose (a) and time (b) dependent cellular uptake. Figure S8. Cellular uptake pathway of PEG-iLNPs (a,b) and PS20-iLNP (c,d). Figure S9. Intracellular distribution of PEG-iLNPs@Cy5mRNA and PS20-iLNP @Cy5mRNA. Figure S10. Fluorescence signal analysis on Laser scanning confocal microscopy images using ImageJ.
