# Supplementary material for: Construction of Spleen-Accumulated Polysorbate 20-Containing Ionizable Lipid Nanoparticles for mRNA Delivery
Source: Nanomaterials (Basel). 2025 Dec 8;15(24):1844. doi: 10.3390/nano15241844 (PMC12735810; doi:10.3390/nano15241844)
Supplement: Supplementary file 1 [file nanomaterials-15-01844-s001.zip › nanomaterials-4012889-supplementary.pdf]

Construction of spleen-accumulated polysorbate 20-containing ionizable lipid nanoparticles for mRNA delivery

Hanyu Liu, Siqi Li, Kexin Chen, Shuyi Yao, Xuefeng Tang*, Xiaojun Han*

State Key Laboratory of Urban-rural Water Resource and Environment, MIIT Key Laboratory of Critical Materials Technology for New Energy Conversion and Storage, Heilongjiang Provincial Joint Laboratory of Molecular Science (International Cooperation), School of Chemistry and Chemical Engineering, Harbin Institute of Technology, Harbin 150001, China

* Correspondence: hanxiaojun@hit.edu.cn

Table S1. Formulation of iLNPs with diverse molar ratio of PS20

Name	SM102 (%)	DOPE (%)	Chol (%)	PS20 (%)
1	50	10	39.5	0.5
2	50	10	39	1.0
3	50	10	38.5	1.5
4	50	10	37.5	2.5
5	50	10	35	5.0
6	50	10	30	10
7	50	10	25	15
8	50	10	10	30

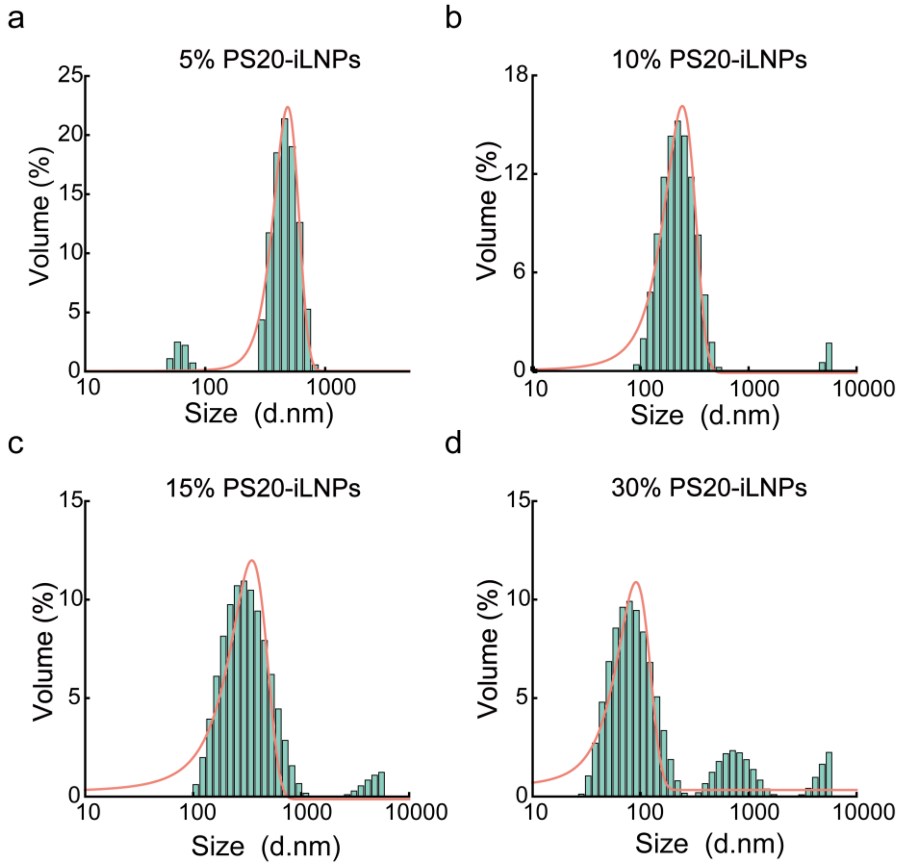

Figure S1. Size distribution of 5% a), 10% b), 15% c), and 30% d) PS20-iLNPs detected by DLS.

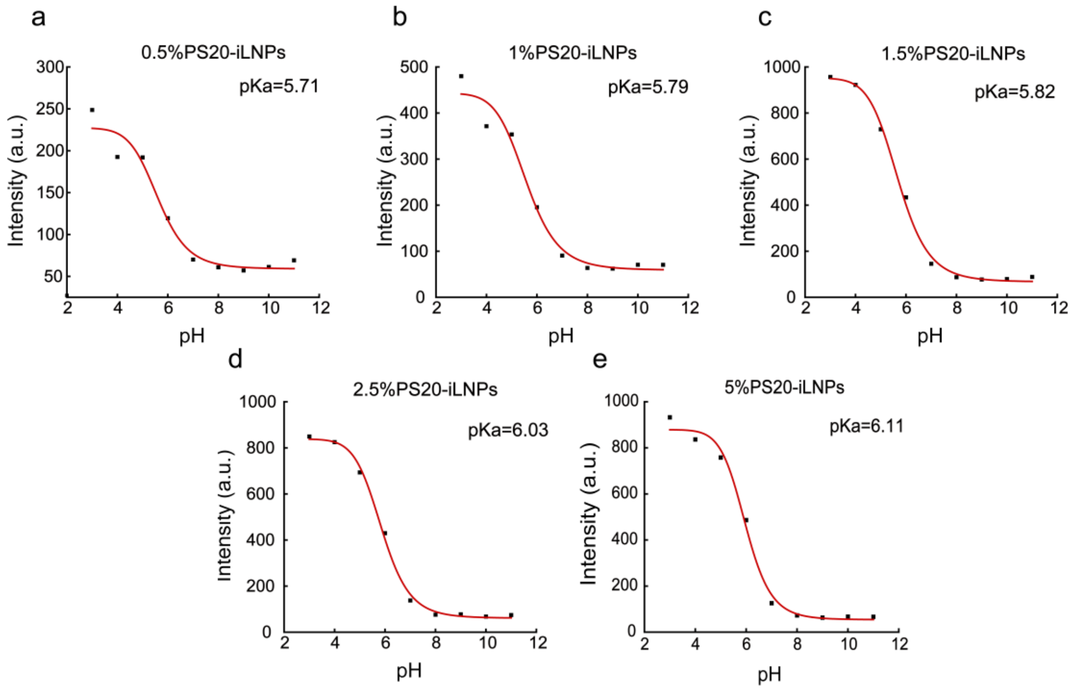

Figure S2. TNS assay. Fluorescence values of 0.5% a), 1.0% b), 1.5% c), 2.5% d), 5.0% e) PS20-iLNPs (0.5-5.0%) at different pH values and S-type fitting curves.

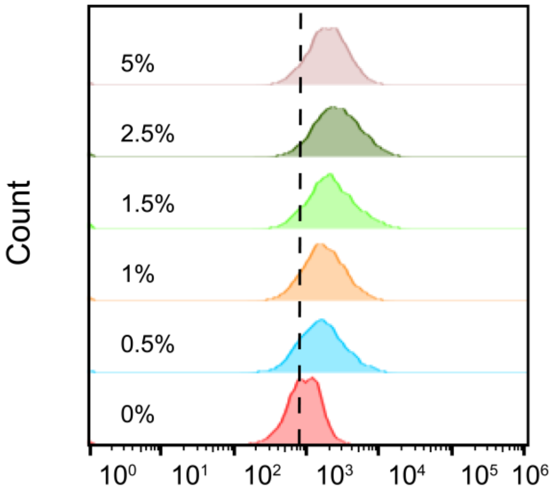

Figure S3. Flow cytometry of C6 cells incubated with iLNPs containing different concentrations of PS20 for 4 h.

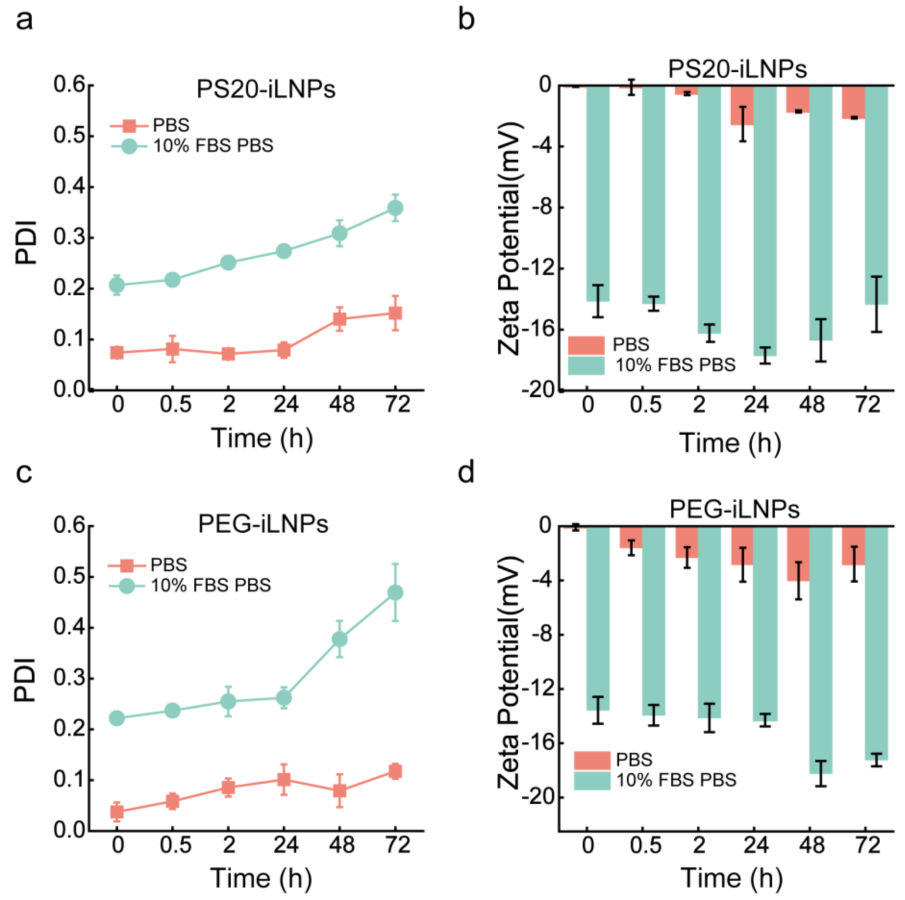

Figure S4. The PDI a, c) and Zeta-potential b, d) of PS20-iLNPs and PEG-iLNPs with different incubation periods characterized by DLS.

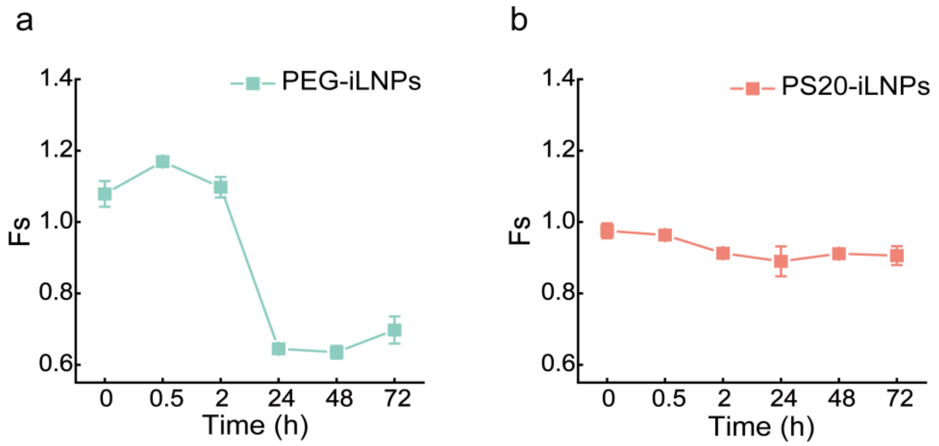

Figure S5. Fs value of PS20-iLNPs a) and PEG-iLNPs b) with different incubation periods.

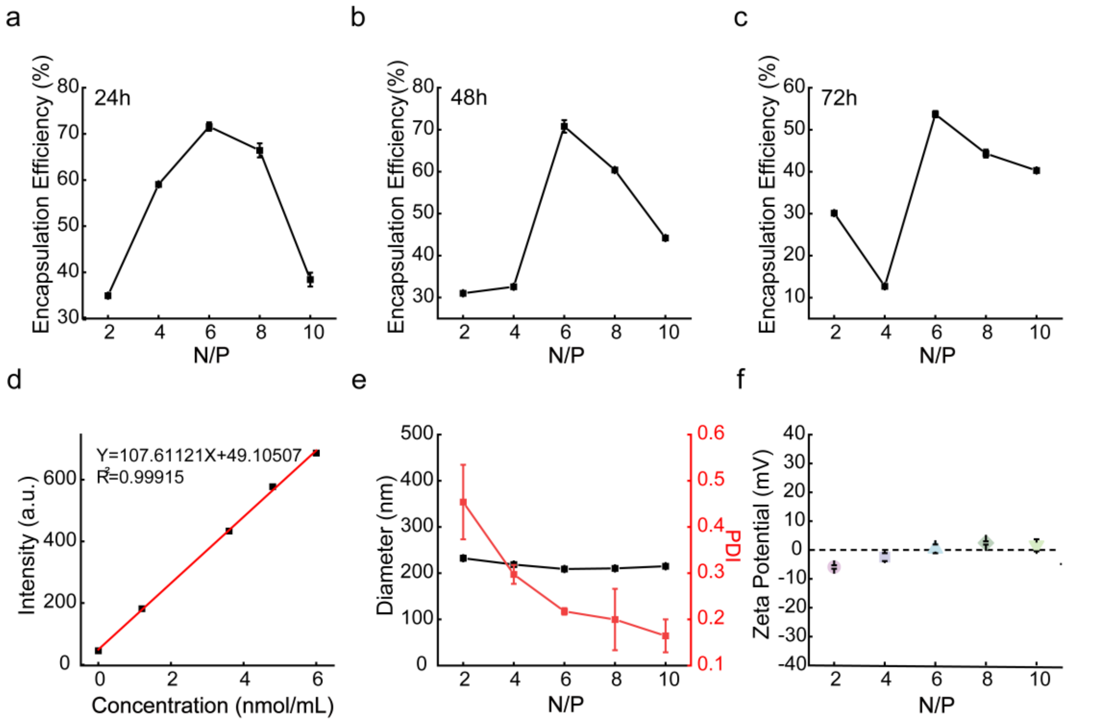

Figure S6. Encapsulation efficiency of PS20-iLNPs@mRNA at different N/P ratios after 24h a), 48h b) and 72h c). d) The calibration curve of TNS assay. e) The size and PDI of PS20-iLNPs@mRNA at different N/P ratios. f) The zeta-potential of PS20-iLNPs@mRNA at different N/P ratios.

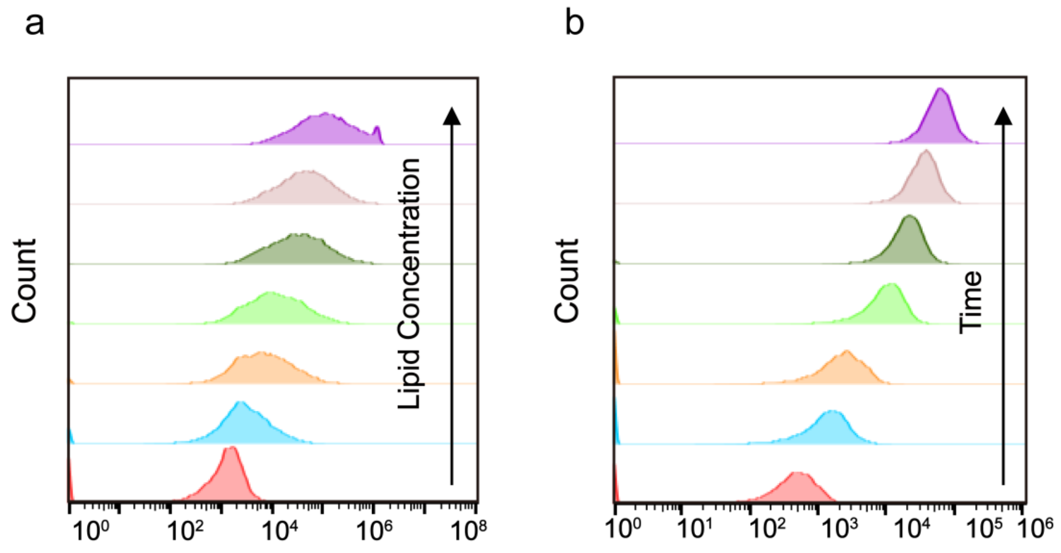

Figure S7. Flow cytometry of C6 cells incubated with iLNPs to assess dose a) and time b) dependent cellular uptake.

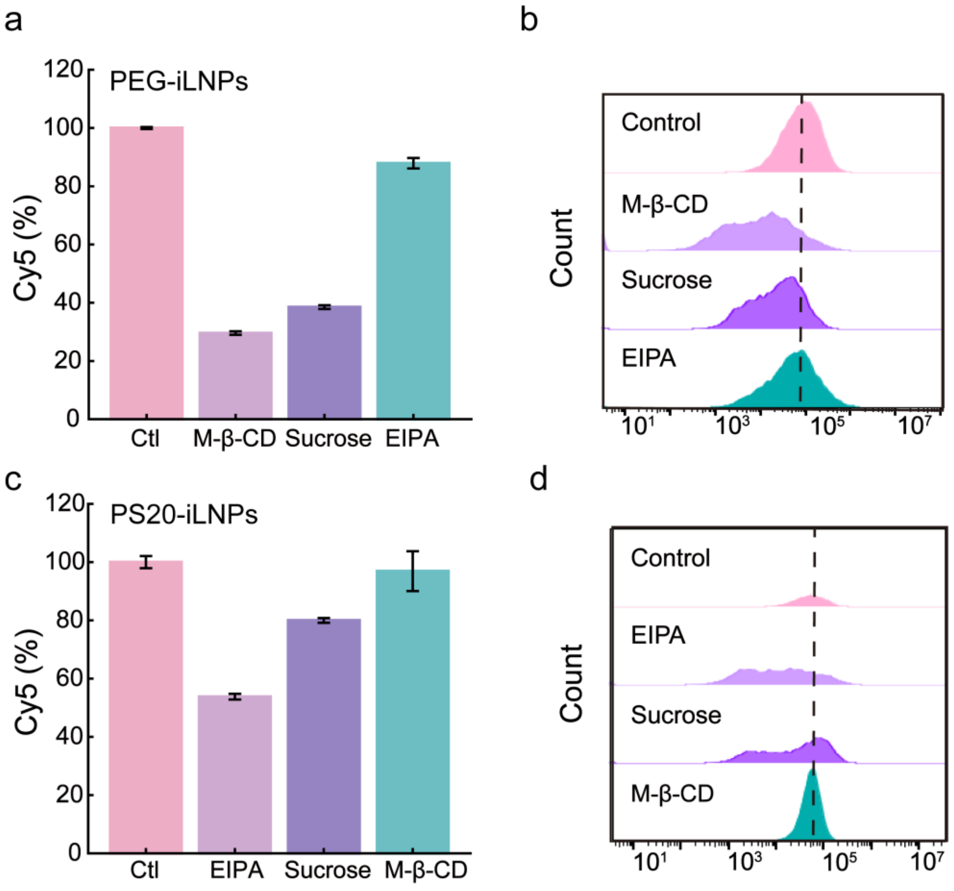

Figure S8. Cellular uptake pathway of PEG-iLNPs a,b) and PS20-iLNP c,d).

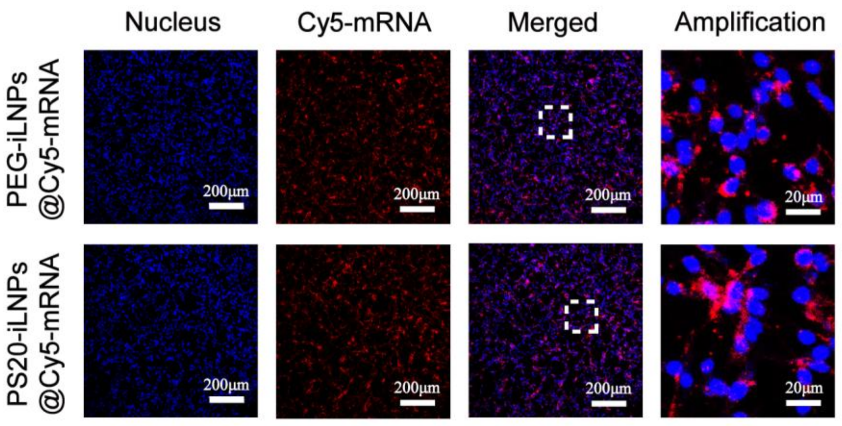

Figure S9. Intracellular distribution of PEG-iLNPs@Cy5mRNA and PS20-iLNP @Cy5mRNA.

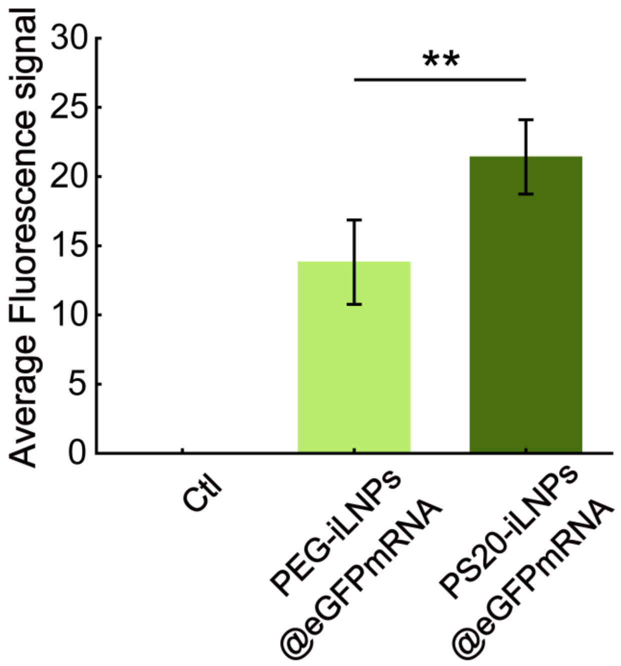

Figure S10. Fluorescence signal analysis on Laser scanning confocal microscopy images using Image J.
